# A survey of knowledge about hepatitis B among new military recruits in China

**DOI:** 10.1186/s40779-016-0110-5

**Published:** 2017-01-18

**Authors:** Yuan-Yuan Li, Wei-Wei Chen, Lei Wei, Yang-Xin Xie, Li-Feng Wang, Jun-Liang Fu, Fu-Sheng Wang

**Affiliations:** Treatment and Research Center for Infectious Diseases, 302 Hospital of Chinese PLA, Beijing, 100039 China

**Keywords:** Hepatitis B virus, New recruits, Infection, Knowledge, China

## Abstract

**Background:**

Hepatitis B is a disease that affects the liver and is caused by the hepatitis B virus (HBV). Hepatitis B is a serious public health problem in China. The objective of this study was to assess knowledge of and behaviours towards the transmission and prevention of hepatitis B of new military recruits in China.

**Methods:**

A cross-sectional study was conducted among 800 new military recruits. A self-administered, structured questionnaire was used to collect information, and 727 questionnaires were returned completed. Analysis was performed using SPSS 18.0, and *P* < 0.05 was considered statistically significant.

**Results:**

Of the respondents, 665 (91.5%) were male and 62 (8.5%) were female. The mean age was 18.9 ± 1.7 years. A total of 608 respondents (83.6%) demonstrated poor knowledge and 119 (16.4%) adequate knowledge about HBV. Older age, female and higher education level were statistically associated with a higher mean knowledge score. Multivariate logistic regression showed that age (*OR* = 3.040, 95%CI 1.724–5.359, *P* < 0.001) and gender (*OR* = 1.791, 95%CI 1.325–2.421, *P* < 0.001) were significantly associated with appropriate behavioural practices towards prevention of HBV.

**Conclusion:**

Against a backdrop of high HBV prevalence in China, new military recruits had poor knowledge of HBV. New recruits need better education about HBV to assist in reducing and preventing HBV infection.

## Background

Hepatitis B virus (HBV) infection is an important global public health problem. Approximately 5% of adults exposed to HBV develop chronic HBV infection [1], and most of the 350 million chronically infected people worldwide are infected in childhood [2]. Approximately 780,000 people die every year from HBV infection; of these, 650,000 die from cirrhosis and hepatocellular carcinoma resulting from chronic infection and another 130,000 die from acute HBV infection [3]. HBV infection is a leading cause of death from liver cancer and cirrhosis [4] and is one of the five most prevalent diseases in mainland China, where approximately 130 million people are carriers of HBV -- almost a third of all people infected with HBV worldwide. Approximately 30 million people in China are chronically infected, with 300,000 dying from HBV-related diseases every year, accounting for 40% to 50% of HBV-related deaths globally [5]. A nationwide HBV sero-epidemiological survey conducted in China in 1992 revealed a hepatitis B surface antigen (HBsAg) carrier rate of 9.8% for the entire population. As a result, the Chinese government initiated a universal HBV immunization programme in the same year, and free HBV vaccination has been provided for all newborns since 2005. These efforts have reduced the HBsAg carrier rate in the general population, from 9.8% in 1992 to 7.2% in 2006 [6]. Although prevention and treatment of hepatitis B has improved, the number of HBV-infected patients in China remains very high.

HBV is highly contagious and is transmitted through parenteral, sexual and vertical (perinatal transmission) routes. An improved understanding of HBV infection routes among the population reduces the risk of HBV infection, which is especially true for young people serving in the military. Young males and females in the military are one group in which it is important to recognize risk behaviours associated with parenterally transmitted diseases. Military personnel often live in camps, predisposing them to exposure to common routes for HBV transmission. Sharing daily utensils, such as hair brushes, combs, razors and toothbrushes, is common among people living in groups and is a behaviour that may facilitate the transmission of viruses [7]. Additionally, soldiers frequently travel for professional reasons and may spend extended periods of time apart from their family. This may encourage soldiers to have multiple sex partners, potentially increasing their risk of exposure to a variety of sexually transmitted infections, including HBV. Military forces operate as a fighting collective, prepared for combat and adaptable to wartime needs. Unremitting effort is required to prevent unnecessary non-combat attrition through the prevention of disease, especially the prevention and control of infectious diseases. In China, the new recruits are not infected with HBV, but we found that recruits still had HBV infection after enlistment, so understanding what influences infectious diseases in military personnel assists in improving and maintaining their health. Our study objective was to develop a questionnaire about basic HBV knowledge and conduct a survey among new recruits in the Chinese army, using the results to inform military health promotion policies and to better prepare personnel for combat-readiness.

## Methods

### Study design, subject selection and data collection

We conducted a prospective cross-sectional survey, using a newly developed questionnaire. Military units that had enlisted recruits in March, 2015 were identified, and a random sample of these recruits was issued a questionnaire for self-completion. A total of 800 copies of the questionnaire were issued. Data were kept anonymous and discarded after the completion of the research.

### Assessing HBV awareness and knowledge

The questionnaire consisted of three sections and was presented in Chinese. The first section focused on the sociodemographic background of the respondents, including their gender, age, marital status, ethnic group, level of education, and type of residential area prior to joining the army. Our definition of “higher education level” indicates a college degree or above, and “older age” indicates 22–24 years old. The second section comprised a set of 15 question-statements exploring knowledge of HBV infection and its consequences, as well as basic knowledge of HBV transmission modes and preventive measures (Table 1). The respondents were then classified as having adequate or inadequate knowledge, using a cut-off score of 9 points or above (i.e., ≥60% correct) to define adequate knowledge. This information was then analysed to examine the factors associated with adequate knowledge. The third section asked about previous hepatitis B vaccination, respondents’ attitude towards people infected with hepatitis B, their source of knowledge about hepatitis B and whether they desire or require more knowledge about hepatitis B.

**Table 1:** Responses of the study participants to basic hepatitis B knowledge items[*n* = (%), *n* = 727]

No.	Statement	Yes	No	Unknown
1	Shaking hands with hepatitis B carriers or talking with them will cause you to become infected with hepatitis B.	87(12.0)	438(60.2)	202(27.8)
2	Do you know any asymptomatic carriers of hepatitis B virus?	100(13.7)	0	627(86.3)
3	Hepatitis B virus carriers may transmit hepatitis B virus to newborns during the delivery process.	315(43.3)	49(6.8%)	363(49.9)
4	Hepatitis B can be transmitted during sexual activity.	262(36.0)	149(20.5)	316(43.5)
5	Close contact with family members can spread hepatitis B virus.	342(47.0)	105(14.5)	280(38.5)
6	Vaccination with hepatitis B vaccine can prevent hepatitis B virus infection.	514(70.7)	25(3.4)	188(25.9)
7	Fever, nausea, vomiting and yellow eyes are common symptoms of hepatitis B.	320(44.0)	350(48.1)	57(7.9)
8	Chronic hepatitis B can develop into liver cirrhosis and liver cancer.	309(42.5)	30(4.1)	388(53.4)
9	Unsanitary dietary habits are not the main transmission route of hepatitis B.	161(22.2)	400(55.0)	166(22.8)
10	Hepatitis B is transmitted by mosquito bites.	180(24.8)	198(27.2)	349(48.0)
11	Many people using the same syringe can spread hepatitis B.	405(55.7)	284(39.1)	38(5.2)
12	The hepatitis B vaccine is the most effective measure to prevent hepatitis B.	571(78.5)	44(6.1)	112(15.4)
13	Vaccination of newborns and 6-month-old infants is included in the hepatitis B vaccine immunization programme in our country.	128(17.6)	91(12.5)	508(69.9)
14	Hepatitis B virus carriers can get married.	332(45.7)	62(8.5)	333(45.8)
15	Hepatitis B virus carriers can give birth to a child.	262(36.0)	92(12.7)	373(51.3)

### Statistical analyses

SPSS 18.0 statistical software was used for data entry and analysis. Descriptive analysis of the sociodemographic characteristics was performed and the results expressed as numbers and percentages. Variables found to be significant in the univariate analysis were included in a multivariate analysis. Logistic regression analysis was used to explore the independent factors associated with HBV knowledge scores. The level of statistical significance was set at *P* < 0.05. Adjusted odds ratios (aORs) and 95% confidence intervals (CIs) were calculated.

## Results

**Fig. 1:**
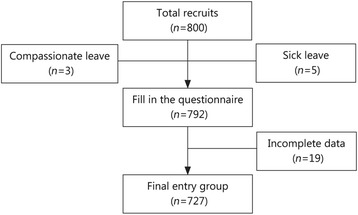
Flow chart showing the screening process for inclusion in this study

Out of a total of 800 questionnaires sent out, 792 valid questionnaires were recovered, giving a response rate of 99.0%. Sixty-five respondents did not complete the basic questionnaire information and were excluded, ultimately leaving a total of 727 completed questionnaires (Fig. 1). Of the 727 respondents, 665 were male (91.5%) and 62 were female (8.5%), with a mean age of 18.9 ± 1.7 years. The majority of the new recruits [663 (91.2%)] aged 16–21 years. 722 (99.3%) respondents were single and 693 (95.3%) were of Han nationality. More than half [406(55.9%)] came from urban areas and 642 (88.3%) had a high school or higher education level.

### Basic knowledge about hepatitis B transmission and prevention

Basic knowledge was assessed by questions focusing on signs and symptoms, transmission and prevention. Each response was scored as ‘yes’, ‘no’ or ‘unknown’. Correct responses were awarded a score of one point, and the total knowledge score, which could range between 0 and 15, was calculated; a knowledge score of <9 was considered as poor and ≥9 was considered as adequate basic knowledge about hepatitis B. The mean score of the entire cohort was 6 ± 2.96. Of the 727 participants, 608 (83.6%) were classified as having poor basic knowledge and 119 (16.4%) showed adequate basic knowledge about HBV. Of the 15 questions, 4 had ‘unknown’ selected by >50% of respondents, and these included questions specifically about the route of hepatitis B transmission and hepatitis B prevention; 627 (86.3%) did not know any asymptomatic carriers of hepatitis B virus and only 262 (36.0%) knew hepatitis B could be transmitted during sexual activity (Table 1).

### Relationship between demographic characteristics and basic knowledge and behaviour scores

The relationship between demographic characteristics and total basic knowledge and behaviour scores is presented in Table 2. Older age, female and higher education level were statistically associated with higher total knowledge scores. The independent factors age (*OR* = 3.040, 95%CI 1.724-5.359, *P* < 0.001) and gender (*OR* = 1.791, 95%CI 1.325-2.421, *P* < 0.001) were significantly associated with appropriate HBV prevention behaviour; the older the respondents (22–24 years), the better their basic HBV knowledge. Females undoubtedly knew more than males about HBV.

**Table 2:** Factors associated with adequate and poor basic HBV knowledge among the population of new military recruits in China [n (%)]

Characteristics	Poor	Good	*P* value
Gender			<0.001
Male(*n* = 665)	569(85.6)	96(14.4)	
Female(*n* = 62)	39(62.9)	23(37.1)	
Age (years)			<0.001
16-18(*n* = 363)	319(87.9)	44(12.1)	
19-21(*n* = 300)	248(82.7)	52(17.3)	
22-24(*n* = 64)	41(64.1)	23(35.9)	
Marital status			0.826
Married(*n* = 5)	4(80.0)	1(20.0)	
Single(*n* = 722)	604(83.7)	118(16.3)	
Ethnicity			0.496
Han ethnicity(*n* = 693)	581(83.8)	112(16.2)	
Minority ethnicity(*n* = 34)	27(79.4)	7(20.6)	
Education level			0.012
Junior high school(*n* = 85)	80(94.1)	5(5.9)	
High school(*n* = 448)	373(83.3)	75(16.7)	
College degree or above (*n* = 194)	155(79.9)	39(20.1)	
Place of residence prior to joining the army			0.663
Large city (municipality or provincial capital) (*n* = 186)	156(83.9)	30(16.1)	
Small city (prefecture- and county-level city) (*n* = 220)	180(81.8)	40(18.2)	
Rural area(*n* = 321)	272(84.7)	49(15.3)	

### Hepatitis B vaccination status

**Fig. 2:**
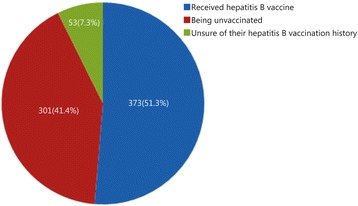
Chart indicating hepatitis B vaccination status prior to recruitment

Of the respondents, a total of 373 (51.3%) had previously received the hepatitis B vaccine, 53 (7.3%) reported being unvaccinated and 301 (41.4%) were unsure of their hepatitis B vaccination history (Fig. 2)

### Attitude towards people with hepatitis B carriers

**Fig. 3:**
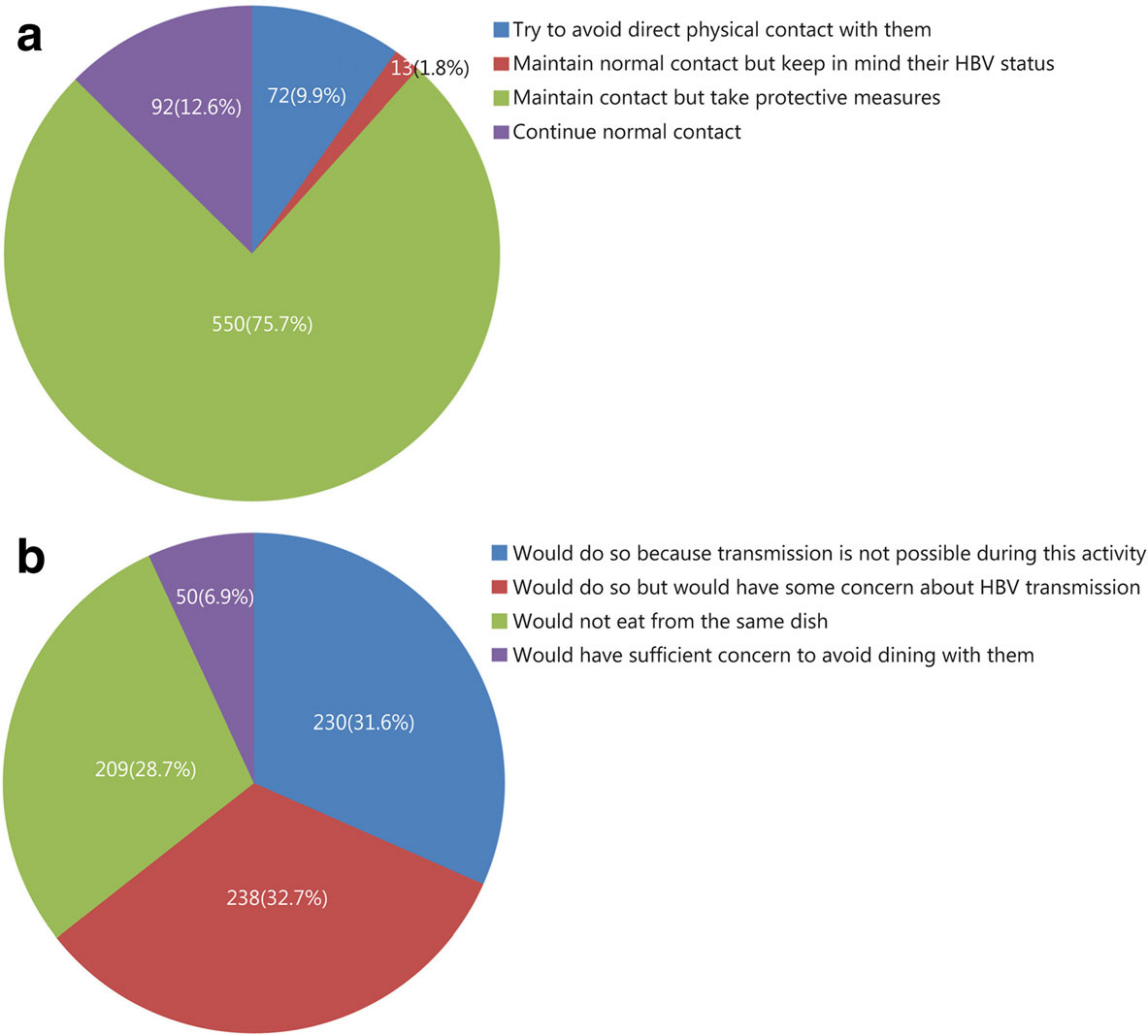
Chart showing the attitudes of respondents to hepatitis B carriers. **a** Contact with hepatitis B carriers; **b** Dealing with hepatitis B carriers

The results revealed that when recruits know that someone around them carries HBV, 72 (9.9%) would try to avoid direct physical contact with them, such as touching, 13 (1.8%) would maintain normal contact but keep in mind their HBV status, 550 (75.7%) would maintain contact but take protective measures, and 92 (12.6%) would continue normal contact (Fig. 3a).

When asked about eating meals with HBV carriers, 230 (31.6%) reported that they would do so because transmission is not possible during this activity, 238 (32.7%) reported that they would do so but would have some concern about HBV transmission, 209 (28.7%) would not eat from the same dish and 50(6.9%) would have sufficient concern to avoid dining with them (Fig. 3b).

## Discussion

Hepatitis B is a highly infectious disease that is highly prevalent in China. Because of the high HBV prevalence, basic knowledge about hepatitis B is conducive to the protection of non-infected people, especially young people, in whom an understanding of hepatitis B is important. In China, military recruits from city areas are required to have a high school or higher education level; in rural areas, this requirement has been relaxed to junior high school. Our survey results reflected this, with high school or higher education accounting for 88.3% of the group, meaning they were mainly around 18 years old. Only 119 (16.4%) of recruits had adequate basic knowledge about hepatitis B. With the introduction of the hepatitis B vaccine, the HBV infection rate has decreased significantly in recent years [6]. However, in rural areas, the HBV infection rate remains high. Screening of 12,393 women of childbearing age in 2013 revealed a positive HBV rate of 9.51% [8]. In the same year, 10.4% of 14,997 cases screened positive for HBV in insular regions of southeast China [9]. Prior to this, an examination of 1050 people in Putian found that 15.8% tested positive for HBV [10]. Military personnel experience greater exposure to transmissible infectious diseases due to the nature of their missions [11, 12]. Being away from home and exposed to several infectious agents makes military personnel more susceptible to infectious diseases, especially HBV, which may influence virus heterogeneity. The army is trained to engage in combat and so military recruits live in collective group environments. If both officers and soldiers paid attention to personal hygiene and increased their knowledge, this would benefit the prevention and treatment of HBV.

The answer ‘do not know’ accounted for 50% of responses for 8 of the 15 questions on basic knowledge of hepatitis B, further illustrating the need to strengthen the dissemination of accurate information about hepatitis B to new military recruits in the future. One study found that a history of surgery, blood transfusion and having hepatitis B-infected family members were high risk factors for HBV infection [13]. Understanding the HBV infection pathway effectively reduces the chances of infection.

Our study found that knowledge of hepatitis B was significantly associated with gender and age. Women demonstrated better hepatitis B knowledge, which may be related to a greater interest in hygiene. The higher level of knowledge among older recruits is more readily understood, as knowledge generally tends to be acquired with age. Ethnicity and former type of residential area had no relationship to hepatitis B knowledge, which suggests that improvement in education about hepatitis B needs to be applied across the population, not just to select groups. Only a clear understanding of HBV transmission routes, symptoms and preventive measures will enable people to be fully involved in hepatitis B prevention.

The army screens potential recruits for HBV and only non-infected people are recruited. More than half of survey respondents had been vaccinated against hepatitis B, with only 53 (7.3%) not inoculated; however, 301 (41.4%) were not aware of their vaccination status. As a result, hepatitis B vaccination will be introduced for unvaccinated recruits as it has been scientifically proven as effective in preventing hepatitis B.

Most recruits reported that they would maintain contact with HBV carriers, but with attention given to protecting themselves, and attitudes towards people with HBV infection were mostly non-discriminatory. However, some results suggested more detailed knowledge of HBV transmission and preventive measures was required, particularly around the issue of dining with HBV carriers. Further emphasis is required about HBV being acquired only through transmission of infected blood and not via the digestive tract, meaning that co-dining is safe.

Certainly, this study has three limitations. Firstly, the survey didn’t mention the recruits’ family income, which may be related with degree of HBV knowledge. Moreover, we could not validate the information by checking with medical records on the self-reported hepatitis B vaccination. Finally, it is a cross-sectional study, cause-effect relationship may be difficult to establish between the factors assessed and HB knowledge.

## Conclusions

Our study suggests that the dissemination of better information regarding hepatitis B to military recruits is imperative. The Chinese Ministry of Health’s “2006 to 2010: China’s hepatitis B virus prevention and control plan” aimed to increase knowledge of prevention of HBV in more than 80% of the population by 2010[14]. However, our research shows that this target remains a long way off. Better education on the prevention of HBV will potentially improve the health of military recruits, and this will eventually positively influence their effectiveness in combat. The simultaneous continued implementation of mass hepatitis B vaccination will further remove HBV from the general population.

## Abbreviations

aOR: Adjusted odds ratio
CI: Confidence interval
HBsAg: Hepatitis B surface antigen
HBV: Hepatitis B virus
